# Is it the real deal? Perception of virtual characters versus humans: an affective cognitive neuroscience perspective

**DOI:** 10.3389/fpsyg.2015.00576

**Published:** 2015-05-12

**Authors:** Aline W. de Borst, Beatrice de Gelder

**Affiliations:** Brain and Emotion Laboratory, Department of Cognitive Neuroscience, Faculty of Psychology and Neuroscience, Maastricht University, Maastricht, Netherlands

**Keywords:** uncanny valley, virtual characters, naturalistic stimuli, virtual reality, fMRI, emotion perception, action perception, social interaction

## Abstract

Recent developments in neuroimaging research support the increased use of naturalistic stimulus material such as film, avatars, or androids. These stimuli allow for a better understanding of how the brain processes information in complex situations while maintaining experimental control. While avatars and androids are well suited to study human cognition, they should not be equated to human stimuli. For example, the uncanny valley hypothesis theorizes that artificial agents with high human-likeness may evoke feelings of eeriness in the human observer. Here we review if, when, and how the perception of human-like avatars and androids differs from the perception of humans and consider how this influences their utilization as stimulus material in social and affective neuroimaging studies. First, we discuss how the appearance of virtual characters affects perception. When stimuli are morphed across categories from non-human to human, the most ambiguous stimuli, rather than the most human-like stimuli, show prolonged classification times and increased eeriness. Human-like to human stimuli show a positive linear relationship with familiarity. Secondly, we show that expressions of emotions in human-like avatars can be perceived similarly to human emotions, with corresponding behavioral, physiological and neuronal activations, with exception of physical dissimilarities. Subsequently, we consider if and when one perceives differences in action representation by artificial agents versus humans. Motor resonance and predictive coding models may account for empirical findings, such as an interference effect on action for observed human-like, natural moving characters. However, the expansion of these models to explain more complex behavior, such as empathy, still needs to be investigated in more detail. Finally, we broaden our outlook to social interaction, where virtual reality stimuli can be utilized to imitate complex social situations.

## Introduction

In the last decade, cognitive neuroscience research and especially studies employing brain imaging methods like functional magnetic resonance imaging (fMRI) and magnetoencephalography (MEG) underwent significant changes in the type of stimulus material used to investigate human cognition. Specifically, this was seen in a shift toward using more naturalistic stimuli, as compared to highly controlled, simplified stimuli. For example, Bartels and Zeki (2004) and Hasson et al. (2004), as well as many subsequent investigations, showed new ways to analyze brain activity arising from complex stimulus material such as video clips or entire films (Bartels et al., 2008; Hasson et al., 2008a,b; Nishimoto et al., 2011; Lahnakoski et al., 2012). The developments have given strong impulse and momentum to the fields of social and affective neuroscience in particular, as these fields may profit significantly from the use of naturalistic stimuli. These stimuli are appealing because they have the benefit of being multi-modal, temporally coherent and engaging, and allow for a better understanding of how the brain processes information in complex everyday situations. Importantly, use of such stimuli provides a means to experimentally control the events and interactions to which the participant is exposed, which is difficult to obtain in real social situations (Tikka et al., 2012).

This recent preference for natural stimuli has not been limited to films, but also extended to the domains of robotics and computer-generated (CG) imagery, enabling interdisciplinary groups to branch between these fields of research. For example, MacDorman and Ishiguro (2006) and Chaminade and Cheng (2009) argued for using human-like robots (androids) in cognitive and social science investigations because of their advantages to study human behavior. Similar to films, which can be seen as controllable simulations of reality, MacDorman and Ishiguro (2006) pointed out that androids could be utilized to simulate social situations in a regulated manner. Especially during social interaction, androids have the advantage of physical presence over CG human-like characters. However, CG characters may also be perceived as lifelike, particularly when presented within an immersive three-dimensional virtual environment. Within this virtual environment participants may experience a sense of being there, called “presence” (Slater et al., 2009, 2010). When experiencing high presence, participants respond in a realistic manner to characters and events in the virtual environment (Sanchez-Vives and Slater, 2005). Therefore, CG characters may be a viable alternative to androids in neuroimaging research, since limitations in laboratory set-up (e.g., restrictions due to the magnetic field) rule out the physical presence of androids during fMRI or MEG measurements. Moreover, CG characters are easier to adapt to experimental requirements, the know-how to construct the characters is more widespread, and the costs are lower. However, in those cases in which interaction between the artificial agent and the participant is not needed, both androids and virtual characters may be presented through videos or images as a less technically challenging approach. Altogether, these stimuli are very well suited to study the brain basis of human cognition in a controlled but natural manner. One can come closer to understanding mental processes taking place during planning, social interaction, decision-making, emotion perception and other real-life situations by simulating these activities with virtual stimuli.

**FIGURE 1:**
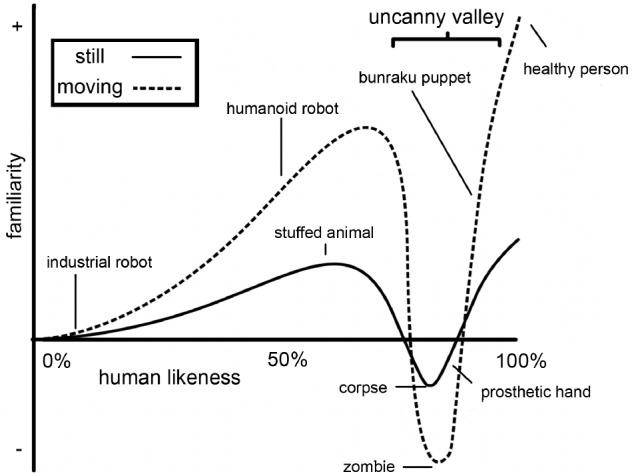
**Illustration of the “uncanny valley” function, shown as a simplified version of the figure appearing in Mori’s (1970) original Energy article from the translation by MacDorman and Minato (MacDorman, 2005)**.

However, aside from the many benefits that using virtual characters as stimulus material in neuroscience research may provide, there may also be pitfalls. On many occasions human-like virtual characters are not just seen as representations of humans, but are treated as equivalent to (photo or video material of) humans. For example, in some studies the implications of using CG faces rather than photographs of faces to study human cognitive processes such as emotion perception are not discussed explicitly (a.o. Klasen et al., 2011). This may be problematic, as human-like characters may evoke different behavioral and brain responses than actual humans (as discussed in work from the same group; Sarkheil et al., 2013). This notion was first illustrated with the uncanny valley hypothesis, formulated by robotics professor Mori (1970). He theorized that characters that resemble humans very strongly, but are not human, cause feelings of eeriness in human observers. The uncanny valley hypothesis claims that when the human likeness of a creature increases, the “Shinwakan” (affinity/familiarity) of the creature increases, until a certain point close to 100% human likeness, where a sharp decrease occurs. This decrease is even more pronounced when the creature is moving (see Figure 1). This valley in the rise of familiarity, which can be described as an uncanny feeling, is called the “uncanny valley.” More recently, this uncanny feeling has been reported during the experience of androids and human-like virtual characters. For example, viewers and actors that were performing alongside the singing human-like robot Geminoid F have described it as eerie and creepy (Waugh, 2012). And the human-like virtual characters in the flopped film production “Final Fantasy: The Spirits Within” were perceived as having “a coldness in the eyes, a mechanical quality in the movements” (Travers, 2001).

However, the differences that may occur between perceiving an artificial agent and a human being are not negative *per se*; they can also teach us more about the conditions under which we still perceive a stimulus as human. In this review we aim to investigate if, when, and how the perception of human-like CG characters and androids differs from the perception of humans, and what factors influence this perception. We approach the matter from a cognitive neuroscience perspective, paying particular attention to the role of virtual characters as potential stimulus material in social and affective neuroimaging studies, and outlining the differences that the perception of these characters may evoke in underlying brain activity when compared to human stimuli. This investigation partly takes the predictions and properties (human likeness and movement) of the uncanny valley into account, but we extend the discussion to other theories and factors relevant in affective and social neuroscience, such as emotion perception and social interaction. First, we consider how the appearance of a virtual character influences its perception, in which categorization plays an important role. Subsequently, we discuss how emotions expressed by avatars and androids are perceived by human observers. We then briefly touch upon the perception of action, where we discuss if and when one perceives differences in action representation by artificial agents versus humans and consider different theories that may explain why one perceives these differences. Finally, we broaden our outlook and see how these processes interact with other social factors during interaction.

## Human Likeness of Virtual Characters

First, we discuss whether the degree of human likeness of virtual characters influences the percept and the feelings they evoke in the observer and whether this gives rise to differences in brain activity. In existing research in this field, human likeness is mainly manipulated through morphing from one image to another in several steps. The human likeness morphing continua generally have one of two endpoints: realistically rendered human-like CG characters (high human likeness) or photographs of humans (100% human likeness). As a starting point a variety of stimuli is used, including non-human CG characters, cartoon characters, robots, or human-like CG characters. The uncanny valley hypothesis predicts that characters with a high human likeness will give rise to a strong sensation of eeriness (Figure 1). MacDorman and Ishiguro (2006) claimed to have found empirical evidence for this hypothesis when comparing ratings of human likeness, familiarity, and eeriness for two sets of morphed photographs that ranged from a humanoid robot to an android to a human. They showed a valley in the familiarity rating for the photographs between the humanoid robot and the android, which was accompanied by an increase in eeriness. However, several subsequent studies have suggested that there might be other mechanisms underlying these results. Cheetham and Jancke (2013) pointed out that not one continuum, but several juxtaposed continua were used in MacDorman and Ishiguro’s study, leading to discontinuities in the human likeness scale. They suggested that one morph continuum with physically equal steps between morphs is a more unbiased way of investigating the relationship between the dimension of human likeness and other factors. Additionally, Burleigh et al. (2013) discussed two alternative hypotheses that may explain MacDorman and Ishiguro’s results: the atypical feature hypothesis and the category conflict hypothesis. The atypical feature hypothesis states that one or more atypical features of the stimulus, such as holes in the forehead that might be typical for a robot but not for a human, together with the level of human likeness account for the perceived eeriness. The category conflict hypothesis suggests that when human likeness of the stimulus is comprised of a morph between two categories (e.g., non-human and human-like) the stimuli in the middle of this scale are perceived as ambiguous, leading to negative affect (Burleigh et al., 2013).

In the first of two studies, Burleigh et al. (2013) manipulated the prototypicality and geometric realism of human-like CG faces in seven equal steps and the participants rated the human likeness, eeriness, fear, disgust, and attractiveness of the faces on 7-point Likert scales. When comparing the subjective human likeness with the perceived eeriness, a linear relation was found between the two, with no presence of an uncanny valley. Similar results were obtained for the relationship between the objective stimulus properties, prototypicality and geometric realism, and the ratings. In a follow-up experiment, the hypotheses mentioned before were tested explicitly, by creating two sets of stimuli. One set manipulated human likeness based on category representation from animal faces to human-looking avatar faces (prototypicality), while the other set varied human likeness on the basis of skin color (blue to natural; realism) for avatars with otherwise human features. An atypical feature (increased eye size) was introduced in both sets. They found that there was a linear relationship between eeriness and subjective human likeness for the realism dataset: when human likeness was high, eeriness was low. The relationship between eeriness and subjective human likeness for the prototypicality dataset was better explained by a quadratic or cubic model, as it showed divergent data points that reflected an increased eeriness around the middle of the subjective human likeness scale. These results supported the category conflict hypothesis. However, the results were not further confirmed by the relationship between objective human likeness and eeriness. Also, no evidence was found for the hypothesis that the combination of the atypical feature with high levels of human likeness increased eeriness.

This categorization conflict has also been investigated in several other studies. Yamada et al. (2013) suggested that categorization difficulty of an ambiguous stimulus leads to higher processing demands and lower processing fluency, which in turn leads to negative affect. They tested morphs between photographs of human and stuffed human, or cartoon human faces (Experiment 1), morphs between photographs of dog and stuffed dog or cartoon dog faces (Experiment 2), and morphs between photographs of different sexes or different identities of human faces (Experiment 3) in forced choice classification and evaluation tasks. The results showed that the most ambiguous images on the realistic versus stuffed/cartoon scales had an increased processing time and showed a decrease in likability, both within and across species. Morphs within one category (sex or identity of human faces) did not show this decrease in likability. The latter results are in line with Burleigh et al. (2013), described above, showing a linear decrease in eeriness with increasing levels of realism (blue to natural skin color) for human-looking avatar faces. This has implications for neuroimaging research in face perception, where within-category morphs are common. These results suggest that no changes in likability should be expected for those continua where human likeness is constant.

In addition to considering stimulus classification speed, Cheetham et al. (2011) also investigated the subjective category boundary for CG avatar to photographic human morphs and measured perceptual discrimination between different sets of these stimuli. They showed that when participants had to classify a stimulus as either human or avatar in a forced choice task, a sharp category boundary was found, showing a stronger categorization than the physical dissimilarity of the stimuli. For example, when a stimulus was physically 33% human, it was categorized as human 10% of the time, while when a stimulus was 66% human, it was categorized as human 90% of the time. Increased classification response times were found for the most ambiguous stimulus at the estimated category boundary, which is similar to the results obtained by Yamada et al. (2013). In the perceptual discrimination task (see also Cheetham and Jancke, 2013), participants were asked to judge sets of two faces (avatar–human, human–human, avatar–avatar), which had an equal distance on the morphing continuum, as same or different. Participants showed a strong tendency to judge sets of faces that crossed the subjective category boundary as different, while this was significantly less for within category changes. This phenomenon of perceiving within-category differences as smaller than between category differences is typically defined as categorical perception and has been shown for different types of stimulus continua (Harnard, 1987; Liberman, 1996; de Gelder et al., 1997; Looser and Wheatley, 2010). A possible explanation of this phenomenon in general has been described in terms of a Bayesian model by Feldman et al. (2009) and is subsequently incorporated into a model specific to the uncanny valley (for more details, see Moore, 2012). In a subsequent set of studies, Cheetham et al. (2014) tested perceptual discrimination with a different task, which used smaller increments between sets of morphs, and employed another analysis method (*d*′ compared to A′ and % different responses). The task (ABX task; Liberman et al., 1957) required participants to judge to which of two previously shown faces a target face corresponded. In the first study, which tested the pattern of perceptual discrimination performance along the dimension of human likeness, discrimination sensitivity of within-category avatar faces was increased compared to across-category faces and within-category human faces. The within-category human faces showed the lowest discrimination sensitivity. In the second experiment, the morphing direction was switched compared to Experiment 1 in order to eliminate possible influences of the morphing algorithm on the perceptual discrimination pattern. In Experiment 2, within-category avatar faces and across-category faces showed increased levels of perceptual discrimination compared to within-category human faces. Participants also rated familiarity of the images, which—in line with other studies—increased with increasing human likeness. Individual variability in perceptual discrimination correlated negatively with familiarity ratings for avatar and ambiguous faces. Finally, Cheetham et al. (2014) investigated in Experiment 3 whether their results could be explained by a differential processing bias, e.g., within-group humans are processed at the exemplar level, while out-group avatars are processed at the category level. This seemed not to be the case, as inversion of the stimuli produced the same results as Experiment 2. Overall, the results indicate that perceptual discrimination was asymmetrical along the human likeness dimension (from human-like to human), with lower discrimination sensitivity for human faces, and that familiarity increased with human likeness. Cheetham et al. (2014) related these findings to fluency amplification (Albrecht and Carbon, 2014): higher levels of fluency (enhanced discrimination) went together with amplified affect (higher feelings of strangeness). However, as processing fluency is broadly defined as “the ease with which information is processed,” various tasks may measure and highlight different aspects of ease of information processing.

Accompanying brain data (Cheetham et al., 2011) suggested that the brain encodes physical and categorical changes of the stimulus differently. The results showed that mid-fusiform regions responded to physical change, while the medial temporal lobe (MTL) and a number of subcortical regions were sensitive for category change. The fusiform face area (FFA) is not only seen as a region particularly responsive to faces, but as responsive to any fine grained distinctions between expertise-acquired categories (Gauthier et al., 2000). Therefore, the activation of fusiform gyrus to the physical change of the morphed stimuli may be understood within this context, as discrimination of face-features depends on experience. Moreover, the physical change in human faces activated a more extensive network of brain regions than physical change in avatar faces. The category change from avatar to human mainly activated the MTL, amygdala and insula, while subcortical regions responded to change from human to avatar. The authors related the MTL activation for human targets to category processing and learning, suggesting that different categorization problems underlie avatar and human target faces. Generally, the subcortical regions and insula that were activated for category change may be related to processing the novelty and uncertainty of across-category stimuli. However, since the insula has been shown to be involved in a wide variety of tasks (ranging from disgust to mental imagery to conflict monitoring), more specific hypotheses should be tested in order to better understand the role of the insula. Moreover, subsequent neuroimaging research could also further validate the other fMRI findings of this study, especially targeting the function of the subcortical regions, such as the thalamus and putamen, in avatar target perception.

In another study Cheetham et al. (2013) examined eye-tracking data during the forced choice categorization task described above. The results confirmed that the most ambiguous stimulus at the subjective category boundary generated the largest conflict in decision making as reflected by increased response times. For ambiguous faces compared to unambiguous avatar faces the dwell time (i.e., duration of the fixation) on the eyes and mouth increased. Thus, the relative importance of facial features changed depending on the category ambiguity. Compared to human faces this did not reach significance.

McDonnell et al. (2012) took both the human-likeness and motion parameters of the uncanny valley hypothesis into account in their study. They created a face-continuum from an abstract cartoon character to a highly realistic human-like CG character. These virtual faces were studied as still images as well as moving images, which were animated using human motion capture. The characters were judged on different aspects, such as realism, familiarity, and appeal. The drop in appeal was not found for the most realistic stimuli, as the uncanny valley hypothesis would predict, but rather for those on the border between cartoon-like and realistic. Consistent with some of the previously discussed studies, McDonnell et al. (2012) suggested that this might be caused by the fact that the characters in the middle of the abstract-to-realism scale may be more difficult to categorize, especially with the mismatch between appearance and motion. Movement of the characters amplified the effects of appeal (higher for appealing stimuli, lower for non-appealing stimuli), which is in line with the uncanny valley hypothesis (see Figure 1). Movement is only one of the properties that can enhance feelings of eeriness when it’s misaligned with the visual appearance of the stimulus. Mitchell et al. (2011) created a cross-modal mismatch between human likeness of a face and the corresponding voice. The incongruent conditions (human face with synthetic voice or robot face with human voice) showed increased ratings of eeriness compared to their congruent counterparts.

Thus, subjective categorization seems to influence processing and experience of stimuli along the dimension of human likeness, when these stimuli are morphed across different categories. This held true for morphing continua from non-human to human-like as well as human-like to human. Ambiguous stimuli at the subjective category boundaries gave rise to prolonged classification response times. However, in other tasks such as perceptual discrimination, processing of ambiguous stimuli may instead be facilitated and differences between avatar and human stimulus processing were observed. In some cases, especially when the morphing continua ranged from non-human to human-like, the most ambiguous stimuli increased eeriness ratings. For human-like to human continua eeriness seemed to decrease linearly. Morphing of faces between identities or genders with equal human likeness showed little changes in likability. However, expertise in human face discrimination among humans may influence processing of human faces versus avatar faces and modulate underlying brain regions (e.g., the fusiform gyrus) that respond to the physical properties of the stimulus. This may have consequences for neuroimaging studies that use avatar faces to study face perception even when comparing avatar conditions directly, as the underlying mechanisms that give rise to enhance or reduced activity to avatar faces are not fully understood. Given the limited number of studies on this subject, an expansion of neuroimaging studies that compare different properties of avatar and human faces and investigate its underlying brain activity in an experimentally and statistically well-constructed way is needed in order to further understand its underlying neuronal mechanisms. Moreover, when designing multi-modal stimuli, the movement and auditory components should match the human likeness (e.g., high human likeness with natural movement and human voice) in order to avoid the sensation of eeriness.

## Perception of Emotion in Virtual Characters and Robots

In the previous section we discussed the feelings that arise from perceiving neutral avatar or human faces with different levels of human-likeness. However, in the field of affective neuroscience the emphasis is not so much on whether neutral stimuli evoke emotions, but rather on how emotional stimuli are perceived. Therefore, we extend the comparison between avatar and human stimuli to emotional faces and bodies.

Several behavioral studies compared the perception of affective human-like avatar faces with the perception of human faces using either still or moving images. For example, Rizzo et al. (2012) compared video clips of facial expressions of emotions by humans or 3D CG avatars. Different types of emotions were expressed, including the six universal emotions (happy, sad, fear, anger, disgust, surprise). Participants were asked to indicate which type of emotion was expressed and how much the clip expressed each of the emotions. The results indicated that the emotions were equally convincing for avatar and human faces. However, the percentage of the clips that were correctly categorized differed for avatar and human video conditions. These results were not very consistent, e.g., sometimes avatars were more correctly identified, while at other times human clips were more correctly categorized. This seemed to depend strongly on the actor used to express the emotion. Another study showed that photographs of human faces and human-like CG avatar faces were recognized comparably well, but that recognition differences occurred for specific emotions (Dyck et al., 2008). For example, disgust was recognized less well, while sadness and fear were recognized better in avatar faces compared to human faces. Thus, behaviorally, recognition of emotions seems to rely more strongly on how (well) the emotions are expressed rather than whether they are expressed by an avatar or human face. However, beyond the explicit recognition of emotions, it is interesting to understand whether emotions expressed by avatars or humans evoke similar patterns in motor and brain responses. First, we review research on facial expressions of emotion within this context and subsequently discuss bodily emotions.

### Motor Responses to Affective Virtual Faces

When observing emotional faces, small responses in the facial muscles of the viewer take place in those muscles that are used for the expression of the emotion. For example, viewing happy human faces is accompanied with electromyography (EMG) activity in the zygomaticus major (ZM), the main muscle for expressing a smile, and viewing angry human faces evokes activity in corrugator supercilii (CS), the muscle for expressing a frown (Dimberg and Petterson, 2000; Dimberg et al., 2000; Aguado et al., 2013). It has been shown that perceiving emotional CG avatar faces results in EMG activity in the same facial muscles as perceiving photographs of human faces, e.g., the ZM for happy avatar faces (Weyers et al., 2006; Likowski et al., 2012) and the CS for sad and angry avatar faces (Likowski et al., 2012). This implies that viewing emotional avatar faces evokes the same muscle responses—called mimicry—in humans as viewing emotional human faces. Dynamic avatar faces (morphed from neutral to an emotion) showed increased EMG activity for happy faces, compared to neutral faces, but this did not extend to angry faces in a study by Weyers et al. (2006). For their avatars the CS activity for dynamic and static angry faces was not significantly different from CS activity for neutral faces. In another study Weyers et al. (2009) showed that these mimicry effects are susceptible to subconscious priming, suggesting that subconscious motives influence empathic mimicry. After priming with neutral words (e.g., street) facial mimicry of emotional avatar faces occurred, but this effect was reduced (less relaxation of CS for happy faces) when the participants were primed with competitive words such as rival or opponent. Likowski et al. (2012) showed that the congruent facial responses between observer and avatar correlated with brain activity in a large network of regions, including inferior frontal gyrus and inferior parietal lobe, regions that have been shown as part of the mirror neuron system. Mirror neurons are neurons that are active during motor execution, but also respond to action observation (Gallese et al., 1996). These results further supported the notion that emotional avatars evoke mimicking behavior in humans and that this is accompanied by activation of brain regions that also activate for the expression of these emotions. In the next paragraph and the section on action perception we will further discuss their role and relevance to action and emotion perception in avatars.

### Brain Responses to Affective Virtual Faces and Robots

We can further investigate the effects of human likeness on emotion perception by looking at accompanying brain activity. Moser et al. (2007) compared brain activity for the recognition of emotions in CG avatar faces versus photographs of human faces. Behaviorally, female participants showed better recognition of emotions in human faces compared to avatar faces in a forced choice task, while males showed no differences. When looking at the brain data for the group as a whole, human and avatar faces evoked similar activity in the amygdala. These results suggest that animated faces may be as effective to investigate perception of emotional facial expressions as human faces. However, these findings are not entirely consistent with the behavioral results, where—at least for women—differences were found in emotion recognition between the two types of stimuli. Therefore, it would be interesting to repeat the experiment with a larger group of subjects, to see if the behavioral differences between the sexes also translate in differential amygdala activation. When comparing the two conditions (human faces versus avatar faces) directly, differences were found in the fusiform gyrus. Previous research has shown that perception of human faces activates the FFA more strongly than other faces, such as animal faces (Kanwisher et al., 1999). The differences in activation found by Moser et al. (2007) might be caused by the fact that the FFA is driven more strongly by within-species faces, as humans are most experienced with classifying human faces. These initial results seem to suggest that although physical differences are perceived between avatar and human faces, the expressed emotions may still be processed in a similar manner.

Opposite effects on the modulation of brain activity in the fusiform gyrus were found for the comparison of emotional facial expressions by a mechanical robot versus a human (Chaminade et al., 2010). Viewing videos of the robot evoked stronger responses overall in visual areas V3, V4, V5, and FFA. Perhaps, because the features of the robot face were so different from the human or avatar face, more visual processing was required to recognize the robot face, leading to enhanced activity in these regions. When looking at the different emotions, emotion-specific activations were found in the insular cortex for disgust and in the right putamen for joy. Although the activations were reduced for the robot emotional expressions, they were not significantly different from the brain responses to the human faces for these emotions. The participants did show enhanced brain activity in orbitofrontal cortex for angry human stimuli compared to angry robot stimuli (which did not differ from baseline). The reduced response to the angry robot stimuli may stem from the fact that the avatar angry faces were rated as significantly less angry than the human angry faces. Chaminade et al. (2010) interpret their results for the insula and putamen in the context of motor resonance, a reaction that has been suggested to rely on mirror neurons. They propose that for viewing human and avatar emotional faces resonance occurs in the observer, which may then play a role in understanding the other person. In the decades since its introduction, the notion of a mirror neuron system at the basis of motor perception has been expanded to explain complex behaviors such as imitation, emotion observation, intention, and empathy (Rizzolatti et al., 2001; Wicker et al., 2003; Iacoboni et al., 2005). While strong evidence has been found for mirror neurons in the context of motor observation, as discussed in the next section, the roles of the regions activated for the experience and observation of emotions and empathy are less clear. For emotions with basic underlying mechanisms such as pain, mirroring properties might hold true, but the more complex the emotional process, the more other mechanisms might come into play. For example, neuroimaging research since the late 19s has shown that when we imagine objects, places or voices, similar regions activate as when we perceive these categories (Cohen et al., 1996; Mellet et al., 1996; Ishai et al., 2000; O’Craven and Kanwisher, 2000; Trojano et al., 2000; Formisano et al., 2002; de Borst et al., 2012). In a sense, imagery also makes the regions involved in perception resonate (Kilner et al., 2007b). As imagery might play a significant role in processes such as empathy, and there is partial overlap between regions attributed to the mirror neuron system and mental imagery networks, it is difficult to attribute the brain activity to mirror neurons *per se*.

In conclusion, for faces looking very dissimilar from human faces, the expressed emotions may evoke reduced responses in the observers, as expressed by lower intensity ratings and reduced brain activity. When emotional avatar faces look highly similar to human faces, they may evoke similar emotional responses as expressed by mimicking responses in the face and activation of emotion regulatory regions. However, differences in brain activity still may occur as a response to the physical differences between avatar and human stimuli. This may be caused by the experience people have with viewing and interpreting human faces.

### Multi-Sensory Integration of Virtual Bodies and Voices

When presenting multi-modal affective virtual stimuli, not only the interaction between human likeness of appearance, movement and voice comes into play, but also the congruency of the expressed emotional content. For multi-modal affective human stimuli, de Gelder et al. (1999) have first shown that affective facial expressions and emotional voice prosody influence each other. A follow-up study by de Gelder and Vroomen (2000a) showed that emotional categorization of facial emotional expressions along a morph continuum from sad to happy was biased toward the emotion expressed in the simultaneously presented voice and *vice versa*. Similarly, Stienen et al. (2011) have shown that emotional human body postures and emotional human voices influenced each other, even when the participants were unaware of the bodies. More recent work has shown that the conscious categorization of ambiguous, affective videos of human bodies (Watson and de Gelder, 2014) and CG human-like bodies (de Gelder et al., 2014) was also influenced by the emotion of human voices. Classification of emotions expressed by CG avatar bodies that were morphed on a continuum from happy to angry, showed an inverted u-shape for response times when participants judged emotion visually. The emotionally ambiguous stimuli showed the largest response times. The categorization curve for emotional avatars showed an increasing percentage of anger responses with the gradual shift from happy to angry. For bi-modal stimuli consisting of a simultaneous CG body and voice expression, voices influenced the rating of the bodily expression in the morphed continuum (de Gelder et al., 2014), consistent with studies on human multi-sensory integration between emotional faces and voices (de Gelder and Vroomen, 2000b; Campanella and Belin, 2007). These initial results on multi-sensory integration of affective virtual bodies and voices indicate that the behavioral effects are similar to those observed with human bodies and voices.

### Emotion Perception in Virtual Reality

Outside of the laboratory, emotion perception often occurs in more complex situations. Some studies have tried to investigate these situations in social experiments. One such example is the famous Milgram experiment on obedience to authority figures, in which participants were instructed to give what they believe are painful electric shocks to another participant each time that participant answers wrongly during a task (Milgram, 1963). Even though performing the shocks gave great distress to the participants, more than half of the participants continued to do so until a final 450-volt shock. Slater et al. (2006) showed that during a 3D virtual version of this experiment participants reacted behaviorally and physiologically as if it were real, even though they knew it was not. Functional MRI evidence showed that individual differences in personal distress during the virtual Milgram experiment co-vary with neuronal changes during perception of the avatar in pain, while no covariance was found with individual changes in emphatic concern (Cheetham et al., 2009). Other life-like responses to virtual reality situations were shown in a bystander study (Slater et al., 2013), in which football supporters were more likely to physically intervene in a confrontation when their attacked CG conversation partner was from the same football club. These results replicate earlier findings from choreographed human situations and illustrate how virtual stimuli can be utilized to imitate complex social situations that might be difficult to orchestrate otherwise.

Evidence so far seems to suggest that expressions of emotions in virtual characters can be perceived similarly to human emotion, with corresponding behavioral and physiological activation. In the brain, evidence for this further accumulates, as emotion-specific regions show similar activation for human-like artificial agents and humans, although physical dissimilarities are also visible. Some typical brain mechanisms, such as multi-sensory integration, seem to influence emotional avatar perception in a manner comparable to the perception of emotions in humans. However, multi-sensory integration is not a phenomenon that occurs only with the perception of humans, but rather is a more general mechanism for integration of sensory modalities in the brain.

## Action Perception in Virtual Characters and Robots

As already mentioned previously, the movement of virtual characters also influences the way in which they are perceived. The interaction between movement and appearance of a human-like stimulus, in behavioral and neuronal effects, has been interpreted in the context of several relatable theories, stemming from different fields. We will briefly discuss these theories and review their empirical support in the current context. The uncanny valley hypothesis, that focusses on behavioral effects, suggests that adding movement increases the familiarity for stimuli that were rated as likeable when still, e.g., for characters with extremes of human likeness on the left and right side of the uncanny valley (see Figure 1). Movement decreases the familiarity even further for human-like images that were rated as unlikeable when still. Comparisons between human movement and avatar/robot movement have also been made in the context of motor resonance. In humans (as well as in the monkey), mirror neurons activate both to the execution and observation of motor actions. Since these neurons are seen as a way to predict and infer actions, robots and virtual characters are quite suitable to study whether our brain only resonates for observing human actions, since these resemble our own motor system, or whether this also occurs for mechanical and CG actions. Some supporters of this theory would predict more resonance (e.g., activity in motor regions) for human-like than artificial action stimuli. In this review, we only briefly touch upon this subject. For more elaborate reviews on the role of mirror neurons and resonance in the perception of androids see Chaminade and Cheng (2009) and Sciutti et al. (2012). Finally, the predictive coding model (Friston, 2005, 2010; Kilner et al., 2007a) suggests that the brain tries to optimize processing at all levels of the cortex, by integrating bottom-up and top-down information through recurrent, reciprocal interactions. At each level predictions are made of the representation in the level below. Through these interactions the error between the sensory expression and its cause is minimized. This framework has been used as a way to explain motor resonance (Kilner et al., 2007a,b). Also, some authors have used this model as an explanation for the uncanny valley interaction of movement and appearance of human-like characters (Saygin et al., 2011). Although predictive coding is well-described for, e.g., action perception and observation, where links between cause (motor goals) and sensory expression (observed kinematics) are relatively direct, generalizing this model to more intricate social phenomena might be more complicated.

### Interaction of Motion and Appearance in Virtual Characters

While the influence of movement on the perception of virtual characters within the uncanny valley hypothesis was confirmed by McDonnell et al. (2012), no such effect was found by Thompson et al. (2011). In their study they manipulated the gait of a human-like CG character and an abstract mannequin CG character based on three kinematic features: articulation, phase, and jerk. The results showed that ratings of humanness and familiarity increased monotonically from least natural to most natural for each of the three kinematic features. An opposite pattern, that is decrease, was found for ratings of eeriness. No differences were found between the mannequin and the human-like avatar. Thus, changes in movement parameters did not show an uncanny valley effect. However, the human likeness parameter was not parametrically adapted in this study. Therefore, it makes these results difficult to compare to those of McDonnell et al. (2012). Ideally, both the human likeness and the kinematic parameters should be manipulated and compared to get a full understanding of the phenomenon. Piwek et al. (2014) manipulated these two parameters and found evidence for the uncanny valley in human likeness, but added motion only increased acceptability of the stimulus, no matter if it was natural or distorted. Their results are in line with Thompson et al. (2011), showing improved familiarity or acceptability when avatars were moving instead of still.

The interaction between appearance and motion can also be investigated in the opposite direction: not the influence of motion on the rating of the appearance, but the influence of appearance on the rating of naturalness of the motion. Chaminade et al. (2007) showed that the response bias to rate a character as biological depends on its human likeness, where higher human likeness coincides with lower ratings of “biological” for both human motion capture data and animated data. However, in this study the degree of human likeness of the virtual characters was not equally spaced. For example, the monster gave a similar response bias to “biological” as the human-like jogger.

### Motion Perception of Artificial Agents in the Brain

Research on the neuronal basis of motion perception has shown differences for avatar or robot motion perception versus human motion perception, as well as congruency effects for combinations of artificial and biological motion with human likeness of the character. For example, the perception of human grasping actions activates the premotor cortex, while the same actions performed by a robot arm do not (Tai et al., 2004). This is in line with motor resonance theory and with results from other behavioral studies (Kilner et al., 2003; Press et al., 2005). These studies showed that executing an arm movement is interfered by observing a human performing an incongruent movement, while this congruency effect does not occur (Kilner et al., 2003), or to a smaller extent (Press et al., 2005) when observing a robot performing an incongruent movement. This effect has been suggested to originate from the velocity profile of biological motion (Kilner et al., 2007c) and interacts with previous experience (Press et al., 2007). However, when the robot has both a human-like appearance and moves naturally, this congruency effect for movement can be found for both robot and human movements (Oztop et al., 2005). These results suggest that when a robot is human-like, motor resonance occurs. In line with previously discussed behavioral findings (Chaminade et al., 2007; McDonnell et al., 2012), an fMRI study by Saygin et al. (2011) showed that when appearance and the expected nature of motion do not match, distinct responses appear in the brain. When investigating repetition suppression in the brain (the reduction of neuronal responses for repeated presentation of the stimulus) for passive viewing of videos with natural human biological motion, videos of robots with artificial motion or videos of humanoids with artificial motion, the largest and most wide-spread suppression effects were found for the incongruent stimulus (i.e., the android with artificial motion), especially in the anterior intraparietal sulcus. This effect seemed to be caused by stronger initial activity (unrepeated stimulus) for the android compared to the robot and human. Saygin et al. (2011) interpret their results on the basis of the predictive coding model, as an increased prediction error in the brain when having to conciliate a human-like character with non-biological motion properties. However, they do not specify how this interaction between properties of different senses (human likeness in the visual domain and naturalness of motion in the motor domain) would be explained by the predictive coding model. The integration across senses and the generation of affective states in the context of the predictive coding model has been discussed more recently in studies on emotion perception, self-representation and multi-sensory integration (Seth, 2013; Apps and Tsakiris, 2014; Ishida et al., 2014; Sel, 2014).

It is fair to conclude that perceived human likeness of a virtual character or robot varies with the naturalness of motion, where high human likeness combined with artificial motion shows an incongruency effect which might be caused by a prediction error in the brain that can be related to higher levels of eeriness experience. The prediction error occurs when two properties of the stimulus do not match and for action perception the prediction error could occur in the mirror neuron system. This suggests that it is important to animate human-like virtual characters and program robots with human-like motion data. Several EMG studies by Huis in ‘t Veld et al. (2014a,b) suggested that specific muscle groups are used for the bodily expressions of emotion in humans. This information, together with motion capture data could be used to improve modeling of biological movements for virtual agents and robots.

## Human–Avatar Social Interaction

The human likeness, naturalness of movement and emotions expressed and evoked by a virtual character or robot are important factors influencing their perception. These and other social factors become particularly relevant when avatars and androids interact with humans. Therefore, in this last section we will go beyond what has been discussed so far and consider implicit processing of virtual characters and robots in order to understand more about social interaction between humans and artificial agents.

In an experiment by McDonnell et al. (2012), participants were asked to tell if a virtual character was lying or telling the truth. There were three CG characters, each rendered differently (cartoon, semi-realistic, highly-realistic) combined with an audio track. As a control, the audio was presented by itself or together with a video of the motion capture session (human). The authors expected that the more unappealing characters would bias the participants toward thinking that they lie more. No such effects were found. The lie ratings and the bias toward believing them were similar for the different renderings and the videos of real humans. However, participants may have extracted most of their information from the audio track that was identical across all stimuli.

When interacting directly with a virtual character, people often show behavior that is similar to human interaction. For example, when offering a gift to a virtual character, participants react much in the same way as they would with humans when this gift is either accepted or rejected (Zucker et al., 2011). When being rejected, brain activity in the anterior insula increases. As discussed previously, the insular cortex has been shown to be involved in a wide variety of tasks, most relating to subjective feelings (Craig, 2009). When the facial expression (happy/disgust) of the virtual character is incongruent with the hand movement (accepting/rejecting), activity in the superior temporal sulcus (STS) rises. This is in line with another study (Vander Wyck et al., 2009) that showed STS activation for viewing of incongruent actions by a human actress (picking up object) based on the emotional context (negative regard), suggesting its role in perception of social acts. Moreover, work by Slater, Sanchez-Vives, and others on virtual embodiment showed that interacting with virtual characters in virtual reality while being embodied in a virtual character gives rise to specific character-dependent changes in behavior, ranging from pain perception to implicit racial bias (a.o. Banakou et al., 2013; Llobera et al., 2013; Peck et al., 2013; Martini et al., 2014). These results indicate that virtual reality stimuli can be utilized to imitate complex social situations and may also affect behavior.

Lucas et al. (2014) even take human-virtual character interaction to another level by suggesting that in some particular cases of social interactions virtual characters might be more successful than real humans. They showed that when disclosing health information, participants were more willing to disclose information to an automated virtual character than to a virtual character controlled by a human operator. They had less hesitancy to disclose information and showed their emotions more openly to the automated virtual character.

Obviously, these interactions are not the only factors influencing how humans perceive artificial agents. Robots and avatars can be programmed to display distinct personalities, and these personalities influence whether they are liked or not. For example, Elkins and Derrick (2013) showed that a virtual interviewer is trusted more when the pitch of the voice is lower during the start of the interview and if the avatar smiles. However, how a virtual characters’ personality is perceived depends largely on context and the personality of the perceiver. When a robot takes on the role of a healthcare assistant, it should have a different personality than when it works as a security guard. People preferred to have an extraverted healthcare robot, showing greater affect, more positive attitudes and greater trust, compared to an introverted robot (Tay et al., 2014). For the security guard however, people showed the opposite preference—it was perceived better, as more trustworthy and more in control when having the introverted personality. The preference for one or the other humanoid personality does not depend only on situation or the task, but also is a function of the participants’ own personality. Extraverts seemed to prefer an extraverted humanoid to encourage them during rehabilitation, while the introverts favored a more nurturing personality (Tapus et al., 2008). Robot or avatar personalities thus may be taken into account when designing stimuli for social neuroimaging experiments. In combination with personality questionnaires for the participant and other hypothesis-relevant measures, personality profiling of avatars may be especially advantageous for virtual reality experiments.

## Conclusion

When designing human-like characters to investigate human cognition in neuroimaging research evidence so far indicates that, contradictory to the predictions of the uncanny valley hypothesis, the most human-like characters are processed most similarly to human stimuli on a behavioral and neuronal level. Thus, not the most realistic looking virtual characters evoke an eerie feeling, but rather those on the border between non-human and human categories, especially if they are combined with human-like motion. This subjective experience seems to arise from difficulty in categorizing ambiguous characters that look neither human nor robot-, avatar- or animal-like, which also leads to increased response times for categorization.

Since humans are experienced in perceiving human faces, viewing avatars may evoke differential processing, e.g., enhanced perceptual discrimination, and modulate underlying brain activity. This suggests that results from avatar and human data may not always be comparable and should be interpreted with care. The underlying mechanisms that give rise to this modulation are not fully understood and therefore further neuroimaging research that compares different physical properties of avatars and humans is needed.

The perception of emotional expressions by human-like characters seems to be fairly similar to perception of human emotions, with corresponding behavioral and physiological activation. This is supported by brain data, although again the physical properties of the stimuli may still cause neuronal differences. For artificial faces that look very dissimilar from human faces, the expressed emotions may evoke reduced responses in the observers, as expressed by lower intensity ratings and reduced brain activity.

Research on the influence of movement on the perceived eeriness of artificial characters shows conflicting results. One study showed the described uncanny valley effect, with an additive effect of motion, while other studies found that added motion only increased the familiarity of the characters. Human-like avatars that move realistically are more likeable and perceived as similar to real humans, as shown, e.g., by the behavioral motion-interference effect and motor resonance in the brain. Non-realistic avatars or robots do not show these effects. Eerie feelings for human-like characters with artificial motion might be explained by the predictive coding model, when the predicted human motion patterns and observed artificial motions lead to an increased prediction error. However, this still needs to be investigated in more detail in order to elaborate the model to more complex processes. It is important in social neuroscience research that, when moving avatars or robots are used, their motion is modeled with biologically appropriate parameters and that possible perceptual differences are taken into account.

When socially interacting with humanoids people may perceive and react as if they were interacting with human beings, showing brain activity in regions relating to emotion and interpersonal experience. Virtual reality experiments may play a significant role in simulating social situations, as these have shown to directly affect social behavior. Neuroimaging experiments could further investigate these virtual experiences by measuring the specific neuronal modulations that lay at the foundation of the behavioral responses.

### Conflict of Interest Statement

The authors declare that the research was conducted in the absence of any commercial or financial relationships that could be construed as a potential conflict of interest.
